# The American Medical Association

**Published:** 1871-01

**Authors:** 


### THE AMERICAN MEDICAL ASSOCIATION.

The Senior Editor of this Journal had the pleasure of attending the last meeting of the American Medical Association, which met at Washington City, in May last.

The Profession was, perhaps, more largely represented than in any preceding meeting of the Association. Marked attention was shown the Association by the resident Physicians, and the high officials of the Government. The occasion was one of great harmony and good feeling, save that a little "disguised politics" was unwisely attempted tc be foisted upon the time and consideration of the body. Politics and medicine are incompatible, and should be severed asunder "as far as from pole to pole." With us, it was a delightful time—a few days of real pleasure. We met many old friends of early days. Pupil and teacher were there to confer and determine what were the best means to advance medical science—to maintain its rights, to sustain its honor, and to perpetuate its institutions. To say that the Association maintained its former reputation in defence of Science, Ethics and its high Educational demands upon the profession, would be superfluous. As long ago as 1857, these principles were announced and strenuously advocated in the Association ; and in this connection it affords us pleasure to reproduce from the pen of the then gifted writer of the Southern Medical and Surgical Journal—Prof. H. F. Campbell of Augusta, Ga.,—his views of the power and influence of this body upon the profession. The learned Professor—now President of the Georgia Medical Association—gave then as a "co-laborer" with us in the Association, our opinions, which we still endorse and maintain. His remarks are as appropriate now as then, and we find them so replete with good sense, and so truthful an expose of the animus of the Association, as well as handsomely written, that we cannot but give them, unmutilated, to our readers :

"An examination of the minutes of the last meeting of the American Medical Association will develope a most active vitality in the association, and an earnest endeavor, on the part of its members to promote the advancement of American Medicine in all its interests, whether *Scientific*, *Ethical* or *Educational*. These three great interests, according to the present constitution of the body, represent the grand triplicate objects of its jealous care. The guarding of these interests are the three great functions of the Association. The first of these functions, every class of the Profession yields most cheerfully to the Association, as its high prerogative and just privilege; at the second, only transgressors of the laws of Ethics are inclined to demur; while in regard to the third, which relates to the reform in Medical Education, there is a class who loudly exclaim against its daring to interfere with the private affairs of institutions, in *whatever* way they may be conducted.

The American Medical Association is not indeed a legislative body—this, its most ardent advocates and firmest defenders will readily admit: It does not pretend to force its dicta as *laws* upon the Profession with the sanctions of ordinary penal codes; but though not a legislative court, it is far from being a body without its legitimate influence, and still further from being devoid of laws, the violation of which are ever atoned for by penalties of the severest kind and most inevitable sequence. In its first establishment, a number of the best, the wisest and most honorable of the Profession, earnestly sought to lay down principles of right, for the direction and guidance of their brethren throughout the country; they met the approbation of the wise and good wherever these principles were read. The principles of right were thus established, and a standard formed by them, which the Profession at large looked to as the criterion, on all questions of propriety pertaining to their conduct.

A standard and a criterion having thus been established, and approved by every one, the power of the Association henceforth rests not so much in themselves as in the hearts and consciences of the Profession at large. It is for the Association only to declare that such and such a measure is not in accordance with the standard of Right, established and acknowledged by them, and by all, in and out of it, and this judgment being pronounced, public opinion applies the castigation which, in time, must inevitably bring reform or confusion to the offenders.

We are aware that there are many who affect to despise the opinions and rail at ethical rules and admonitions of the American Medical Association, as arrogant, offensive, and based on a power only ideal. Berkley asserted, and believed, that life was *all a dream*, and that external objects and the powers of nature were but the ideal *representations* of things—yet Berkley carefully avoided getting into the fire, for fear of being burned, and as carefully shunned the sea, for fear of being drowned. So these pretended despisers of the Association, affecting to contemn its opinions and its judgments, yet are ever anxious to prove that they transgress none of its rules, but have squared with the last letter of its counsels.

The American Medical Association then *is* potent—it has a *moral* power rooted deeply in the hearts and consciences of men, and ramifying throughout the length and breadth of this wide extended land,—it is the true exponent, the highest tribunal of right in the Profession. It has only to be true to itself and to its own great principles—keeping these ever before the world as the *standard of Medical Ethics*, never swerving from them in one jot or tittle, but on the other hand, avoiding all petty and meddling tyranny towards particular individuals or particular institutions —and thus, however much some may affect to despise its admonitions and to beard its power, still reform will follow in its train, —for as the fire *will* burn and the sea *will* drown, so will the violation of its ethical laws wither and overwhelm all who may be hardy enough to continue in transgression."

The above has our unqalified endorsement. The truth of today will he the truth through all time. To the lawless and forgetful we will say—as a check to desperation and self-destruction —heed the voice of reason! The power and influence of professional ethics, founded upon truth and justice, is as potent now as they were in 1857—*a verbis legis non est recedendum.*

